# Supplementary material for: Haplocauda, a New Genus of Fireflies Endemic to the Amazon Rainforest (Coleoptera: Lampyridae)
Source: Insects. 2022 Jan 5;13(1):58. doi: 10.3390/insects13010058 (PMC8778453; doi:10.3390/insects13010058)
Supplement: Supplementary file 1 [file insects-13-00058-s001.zip › Supplementary Materials S1.pdf]

Supplementary Material S1. Material examined of the outgroup taxa.

Amydetinae

Amydetes fastigiata. **Brazil.** *Rio de Janeiro*. Niterói (São Francisco), 1 male, 24.VI.2012, E. Matos, col. (DZRJ)

Psilocladinae

Psilocladus miltoderus. **Brazil.** *Amazonas*. Manaus, Reserva Ducke, 9.ix.1986, 1 male, B.C. Klein col. (INPA).

Lampyrinae

Costalampys tricolor. Holotype: BRAZIL: 1♂, #292 (MNHN, col. Gorham; formerly in col. Chevrolat col). Other material examined. BRAZIL: Minas Gerais: 1♀, Bocaina de Minas, Ribeirão Santa Casa, prox. Cachoeira da Raposa, 22°18′24.7″S, 44°33′54″W, 1294 m, M&M col. (DZRJ); 1♀, Itajubá, R.B.M. Serra dos Toledos, coleta diurna, 08.XI.2015, Rosa & Ladenthin col. (DZRJ); 1♂, 16.II.–17.III.2017, Rosa & Lopes col. (DZRJ); 1♀, São Roque de Minas, P.N. Serra da Canastra, Malaise trap, Mata ciliar, Ponto 1, 3♂, 14–19.XII.2013, Melo & Rosa col. (DZRJ). Rio de Janeiro: 1♂, Angra dos Reis, PE Ilha Grande, Malaise trap, P450A (23°08′47.2″S, 44°11′09.4″W), 441 m, IX.2017, L. Campello, L. Silveira & R. Queiroz col. (DZRJ); 1♀, Itatiaia, P.N. Itatiaia, PENSARIOP2 (22°25′59,6″S, 44°37′39,7″W, 1280 m), Malaise trap, I.2015, R. Monteiro col. (DZRJ); 1♂, 1♀, Teresópolis, P.N. Serra dos Órgãos, 03–05.XI.2014, 1200 m, active search [afternoon], L. Silveira col. (DZRJ). Paraná: 1♂, Curitiba, 28–XII.1976, V. Graf col. (DZUP); 1♂, Curitiba, 12.69, Mielke col. (DZUP); 1♀, Tijucas do Sul, Vossoroca, 25.I, Pe. Moure & Marinoni col. (DZUP); 1♀, Ponta Grossa, Jardim Carvalho, 25°4′39.15″S, 50°9′24″W, 05.I.2014, Nascimento, E.A. col. (UECO); 1♂, Ponta Grossa, Parque Nacional dos Campos Gerais, Morro do Castelo, 25°6′13.90″S, 49°56′36.38″W, 15.I.2014, Nascimento, E.A. & eq. col. (UECO); 1♂, 1♀, Ponta Grossa, em copula [in copula], 12.55, M. Vilella col. (DZUP); Rio Grande do Sul: 1♂, 1♀, Caxias do Sul, Vila Oliva, in copula, 19.2.49 (MAPA); 1♂, Esmeralda, 12.XII.1978, C. J. Becker leg. (MCZ).

Costalampys delicata. Holotype: BRAZIL: Rio de Janeiro: 1♂, Teresópolis, P.N. Serra dos Órgãos, Malaise trap, PVE9B (22°26′57.8″S, 43°0′13.7″W, 1,236 m), I.2016, L. Silveira col. (DZRJ). Paratypes: BRAZIL: Rio de Janeiro: 1♂, Paraty, P.N. Serra da Bocaina, Estr. Paraty-Cunha, 1514 m, 23.I.2010, Mattos, I. & Mermudes col. (DZRJ); 1♀, Teresópolis, P.N. Serra dos Órgãos, Trilha da Pedra do Sino, Cach. Véu da Noiva, 29–31.I.2014, active search [morning ~10am], L. Silveira col. (DZRJ); 1♀, P.N. Serra dos Órgãos, I.2014, PENSARIOP2 (22°26′48″S, 43°00′42,6″W, 1050 m), 1♂, Malaise trap, R. Monteiro col. (DZRJ); Teresópolis, P.N. Serra dos Órgãos, Represa Beija-Flor, 14–17.I.2015, active search [afternoon ~3pm], L. Silveira col. (DZRJ); 1♂, P.N. Serra dos Órgãos, Malaise trap, PVE7B (22°27′24,8″S, 42°59′7,2″W, 952 m), I.2015, L. Silveira col. (DZRJ); 1♂, 2♀, PVE9A (22°26′55,1″S, 43°00′16,4″W, 1246 m), XII.2015, Silveira & Khattar col. (DZRJ); 1♀, Teresópolis, P.N. Serra dos Órgãos, I.1969, Porter & Garcia col. (DZUP).

Costalampys klugii. Holotype: BRAZIL: 1♂, Without other data (ZIN). Other material examined: BRAZIL: 2 males and 2♀, Without other data, #31532 (ZMB); Santa Catarina: 1♂, Seara [Nova Teutonia], XII.1935, B. Pohl. col (MZSP).

Cladodes flabellatus. **Chile.** Without other provenance data. 1 Male. (BMNH);

Dadophora hyalina. **Brazil.** Without other provenance data. 1 Male. (BMNH);

Dilychnia guttula. **Brazil.** *Amazonas.* São Paulo Olivença, 10.VIII.1925, 1 female and 1 male, H.L. Boy (MNRJ).

Ethra marginata. **Brazil.** *Rio de Janeiro.* Teresópolis, without date, 1 male (DZRJ).

Lucidota banoni. **French Guiana.** Without other provenance data, 1 male, 1 female (DZRJ).

Luciuranus josephi. **PARATYPES. Brazil.** *Rio de Janeiro.* Teresópolis, Serra dos Órgãos N. P., 1961m, VIII.2014, 1 male and 1 female, L. Silveira e G. Khattar col. (DZRJ).

Scissicauda disjuncta. **Brazil.** *Rio de Janeiro.* Teresópolis, Serra dos Órgãos N. P., 1050m, XII/2013, Malaise trap, 1 male and 1 female, R. Monteiro col. (DZRJ)

Scissicauda balena. **HOLOTYPE. Brazil.** *Espírito Santo*, 1 male, Descourtils col., coll. Fry (BMNH).

PARATYPE. Idem, 1 female. (BMNH).

Uanauna angaporan. **PARATYPES. Brazil.** *Rio de Janeiro*, Itaguaí, Parque Estadual Cunhambebe, 22º 50′ 53.4″ S 43º 54′ 29.3″ W, 150m, IX.2011, 1 male L. Silveira, Clarkson Sampaio & Ferreira-Jr col. (DZRJ); Angra dos Reis, Parque Estadual da Ilha Grande (PEIG), approx. 23º 10′ 30.4′′ S 44º 11′ 11.3′′ W, IX.2008, 2 females, Projeto Coleoptera [J. Mermudes] col. (MNRJ).

Ybytyramoan praeclarum. **PARATYPE. Brazil.** *Rio de Janeiro*, Teresópolis, P.N. Serra dos Órgãos, Pedra do Sino, Abrigo 4, 23.IX.2011, 1 male, N.C., C.L., J.C., Manuella Folly col. (DZRJ).

Incertae sedis

Araucariocladus hiems. **PARATYPE**. **Brazil**. *Rio de Janeiro*. Teresópolis, Parque Nacional da Serra dos Órgãos, VI.2013, S 22º 27′ 34.6″, W 43º 01 40.2″, 1824m, R. Monteiro col., 1 male (DZRJ).
